# Transparent heater with meshed amorphous oxide/metal/amorphous oxide for electric vehicle applications

**DOI:** 10.1038/s41598-020-66514-8

**Published:** 2020-06-16

**Authors:** Sang Yeol Lee, Jin Young Hwang

**Affiliations:** Department of Semiconductor Engineering, Cheongju University, Cheongju, Chungbuk 360-764 Republic of Korea

**Keywords:** Electronic properties and materials, Structural properties

## Abstract

For electric vehicle application, one of the problems to be solved is defrosting or defogging a windshield or a side mirror without gas-fired heaters. In this paper, we report on a high performance of transparent heater with meshed amorphous-SiInZnO (SIZO)/ Ag/ amorphous-SiInZnO (SIZO) (SAS) for pure electric vehicles. We have adopted amorphous oxide materials like SIZO since SIZO is well known amorphous oxide materials showing high transparency and smooth surface roughness. With the mesh processing technology, a transparent electrode with high transmittance of 91% and low sheet resistance of 13.8 Ω/ϒ was implemented. When a 10 V supply voltage is applied to transparent heater, the transparent heater on glass substrate was heated up to 130^o^C in just 5 seconds and then reached to 250^o^C after tens of seconds due to the low sheet resistance. In addition, the SAS transparent meshed heater (TMH) showed high stability under cycling test and long time working stability test.

## Introduction

In the context of rapid development of Internet of Things (IoT) and 5 G communications, the gas-free electric vehicle is once again became a main topic of recent research. In order to prepare for the era of electrified transportation, the researcher has focused on the auto parts technology that is used by fuel-free electric vehicles, for example, the transparent heater applied in defrosting or defogging a windshield or a side mirror in the winter^1^.

High transparency in visible region and high electrical conductivity are two important factors of transparent heaters, and this technology is based on the studies of transparent conductive electrode (TCE). Compared with the TCEs that are used for display applications or solar energy applications, for the purpose of rapid heating, transparent heaters are suggested to have better electrical conductivity^2^.

Electrical properties of TCEs are usually evaluated by measuring the thin films’ resistivity, which could be characterized by the reciprocal product of carrier concentration and mobility^3^. For instance, In-Sn-O (ITO) is the dominant material in the TCE industry and also be used in the recent manufacturing process of the side mirror^4^. The standard electrical properties of ITO thin films, such as carrier concentration, mobility and corresponding resistivity are about 10^20^ cm^−3^, 10 cm^2^/V·s and 10^−4^ Ω·cm in magnitude^5^. It can be seen from the previous studies of TCEs that an ideal method to improve the conductivity of TCE thin films is to limit the carrier concentration and increase the carrier mobility simultaneously^6^. Nevertheless, until now, it is too difficult to improve the mobility to 10^3^ cm^2^/V·s in magnitude by using the conventional TCE materials^6^: for example, transparent conductive oxides (TCOs), such as ITO, aluminum-zinc-oxide (AZO)^7^, gallium-zinc-oxide (GZO)^2^. Their mobility will be limited as a result of carriers scattering when carrier concentration is greater than 10^20^ cm^−3 ^^8^; otherwise, nanowire networks such as Ag nanowire (Ag NW)^9^, carbon nanotube (CNT)^10^, whose electrical conduction path is confined by geometrical shape or direction of the nanowires. So far the reported high conductive films with resistivity below 10^−5^ ohm·cm always have high carrier concentration above 10^21^ cm^−3 ^^6^.

Meanwhile, carrier concentration is also a very important parameter of optical properties of TCE. The fact is: when light is raying on the TCE thin films with high free carrier density, the electromagnetic wave – free electron scattering, known as phonon scattering becomes the dominant reaction, according to the research of Boltasseva’s group^11^, the optical properties will act as metal-like materials when the carrier concentration is over 10^20^ cm^−3^, which means there will be a high optical loss in visible and infrared region.

Research into metal oxide-metal-metal oxide (OMO) based on the TCEs has lately been progressing by many groups^12,13^. Some major advantages of multilayer structured TCEs, which benefits from the inserted metal layer, includes high transparence, low resistivity, enhanced mechanical property, and lower overall thickness, etc. Besides, as the development of the roll-to-roll is growing, OMO based TCEs start to attract more attention in recent years^14^. However, OMO based TCEs are still limited to the mutual restraint relationship between electrical and optical properties. Thickening the metal layer will effectively improve the conductivity of OMO based TCEs, whereas the thickened metal layer will result in an increase in optical loss^15^.

Grid or mesh processing technology is a method of processing the opaque metal films into micron scale lines which is difficult to be observed with the naked eye of human beings, and therefore ensure the film become transparent^16^. In previous researches, the SIZO has reported some advantages to apply for transparent heater, such as amorphous structure in room temperature, high transparency and smooth surface roughness^16–18^. The smooth surface roughness prevents scattering of incident light, thereby obtaining high transmittance^19^. We investigated the electrical properties and optical properties of SIZO/Ag/SIZO (SAS) transparent mesh heater (TMH). In order to maintain the electrical conductivity while increasing the optical transparency of these SAS structures, with the help of the mesh processing, the SAS TCEs are formed into mesh shapes by using mesh pattern mask, and these mesh-shaped SAS TCEs show low resistivity at the level of 10^−4^ Ω·cm, and high transmittance in visible region above 90%. Mesh-type SAS structure using amorphous oxide shows lower resistance than other references^20^. Therefore, it has advantage to heat up to high temperature even at low voltage and to have long-term working stability.

## Results and Discussion

**Figure 1:**
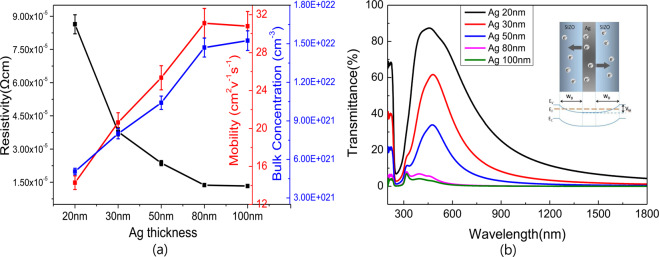
(**a**) Resistivity, mobility and carrier concentration of as deposited SAS multilayers measured by Hall measurement, and (**b**) optical transmittance curves of SAS multilayers, the insert schematic shows ohmic contact formation and carrier diffusion between SIZO and Ag layer in SAS structure.

Before mesh process, as-deposited SAS multilayers samples were fabricated with different Ag thickness from 20 nm to 100 nm while the thickness of top and bottom SIZO layers was fixed as 80 nm. The electrical resistivity of these samples is measured by HALL measurement. In this method, resistivity of thin films is determined by two parameters, bulk concentration and free carrier mobility, as following^3^:

1 $${\rm{\rho }}=1/{\rm{ne}}{\rm{\mu }}$$

where ρ is the resistivity, n is the number of charge carriers, e is the charge of the carrier, and μ is the carrier mobility. As explained in previous research, in an OMO multilayer system, middle metal layer plays the role of carrier injection layer which provided most movable electrons^21^. Work function of semiconductor SIZO is measured to be in the region of 4.6~4.7 eV^22^, and silver layer is 4.4 eV^23^. Since SIZO is an n-type degenerated semiconductor, it is easy to form an Ohmic-contact with Ag layer, then free carriers will diffuse from Ag layer to both top and bottom SIZO layers, as shown in insert inset schematic in Fig. 1b. As shown in Fig. 1a, the carrier concentration of OMO multilayers increased linearly from 5.058×10^21^ cm^−3^ to 1.524×10^22^ cm^−3^ as the thickness of Ag layer was increased. Meanwhile, mobility values of these multilayers also increased from 14.29 to 31.1 cm^2^/V·s, which can be interpreted that conductive path of Ag is mainly formed by the large 5 s orbits of Ag atoms, where free electrons can moved smoothly, therefore mobility of Ag thin film have a relative high value above 32 cm^2^/V·s^6^. It is also observed that mobility would get close to the level of Ag single layer gradually, when Ag components increased in an Ag-SIZO composite structure. Based on Eq. 1, the resistivity is decreased from 8.64×10^−5^ Ω·cm to 1.33×10^−5^ Ω·cm as increasing carrier concentration and mobility. As a result, increasing the thickness of Ag layer will effectively improve the conductivity of the SAS multilayers.

However, from the optical transmittance curve as shown in Fig. 1b, it is observed that the transmittance is gradually lowered as increasing Ag layer thickness: plasma wavelength shifted to short wavelength part because the carrier concentration of SAS multilayer is getting closer to the level of the Ag single layer^24^, due to the increase of the Ag thickness as shown in Fig. 1a, which results in the degradation of optical transmission window. If the thickness exceeds 80 nm, which is thicker than skin depth of Ag^24^, the transmittance of the multilayer in visible region is sharply reduced to 2.1%, and finally shows the bulk-metal-like optical property, known as specular reflection.

**Figure 2:**
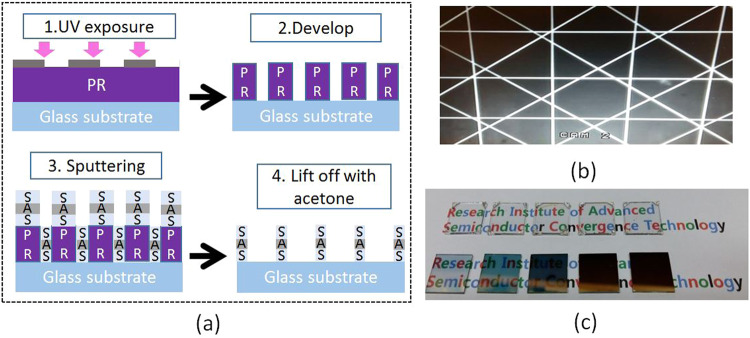
(**a**) Schematic illustration of the mesh patterning process, (**b**) Microscope images of the SAS multilayer meshes on glass substrate, and (**c**) picture of fabricated as-deposited SAS multilayers (bottom row), and corresponding meshed SAS electrodes (top row) with varied middle Ag thickness from 20 nm to 100 nm.

To fabricate a highly transparent electrode while keeping the electrical conductivity, meshed SAS electrodes were designed by taking the use of lithography processing. Over waste of Ag, which can be caused by lithography, can be prevented by various deposition methods^25,26^, such as printing process, electrodeposition, self-assembling Ag nanoparticles and nanowires inks and etching process. To avoid the use of intensely corrosive acids in the lithography process, negative photoresist was chosen and the process details were shown in Fig. 2a. Figure 2b is a microscopic image of processed mesh electrode fabricated by this method, it can be seen that both the mesh lines and the intersections were smoothly formed. Figure 2c shows a picture of SAS multilayers before and after meshing process, and as-deposited samples were listed in the bottom row, with middle Ag layer of 20 nm, 30 nm, 50 nm, 80 nm, 100 nm, from left to right. As the Ag thickness is increasing, the colors of these samples are changed from light blue to dark grey, and finally to a mirror-like if thickness of Ag is over 80 nm. Therefore, in order to increase transparent and maintain low resistance, a mesh type process is applied. After mesh process, all the samples showed high transparency as shown in the top row, because the width of these mesh lines is only 15 μm which cannot be observed by eye.

**Figure 3:**
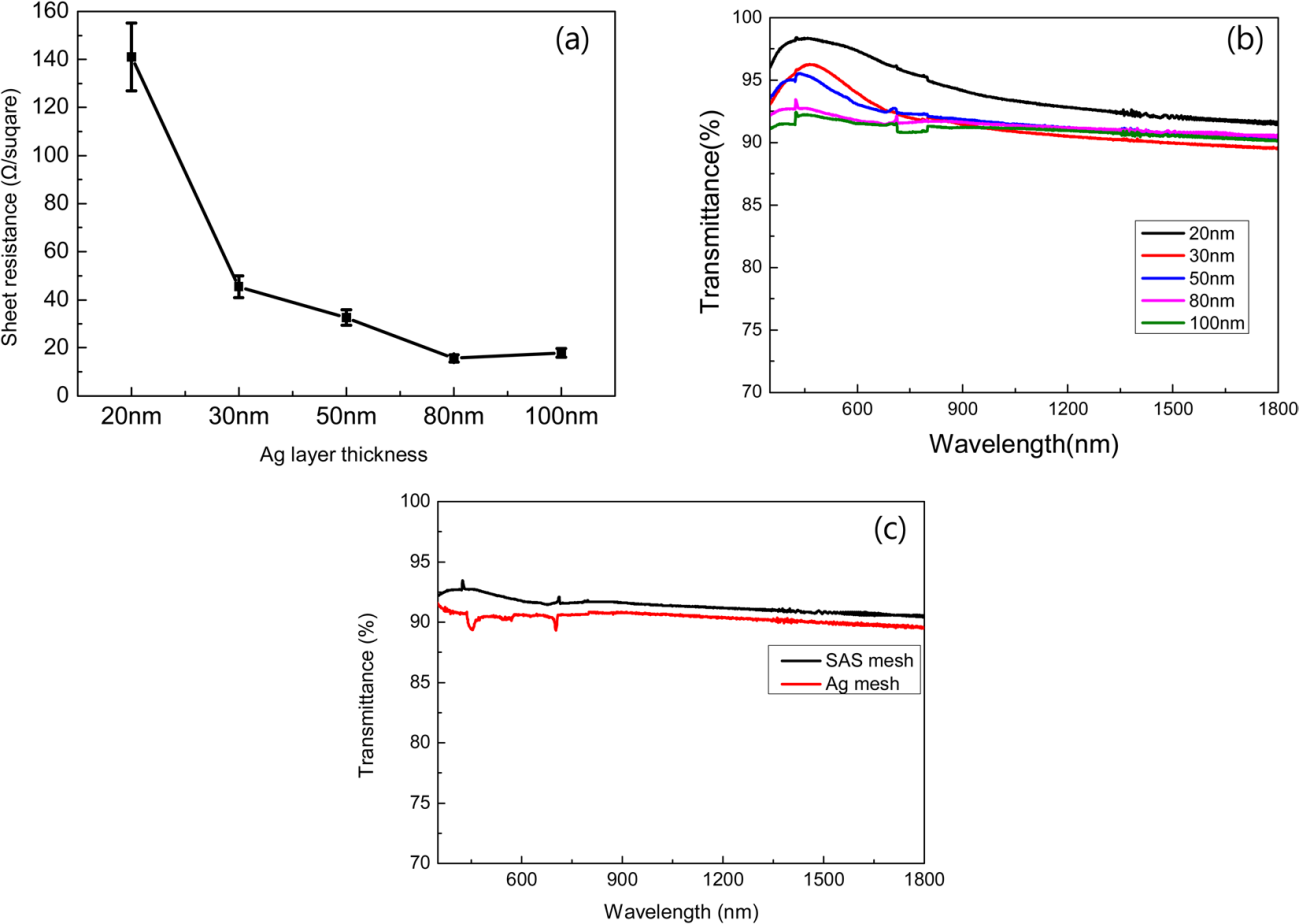
(**a**) Sheet resistance of meshed SAS multilayers with varied thickness of middle Ag layers, and (**b**) optical transmittance curves of these meshed SAS electrodes, and (**c**) comparison of transmittance between SAS mesh and Ag mesh.

Figure 3a shows the electrical properties of these meshed SAS structures. In the case of the layered SAS, the current flows through whole layers. But in the case of the meshed SAS, the high resistivity is measured since the current flows only along a meshed line. Obviously, conductive path of full covered SAS multilayer will be superior to meshed electrode, but take consider of transparent heater using for large-area car window, the separate electric paths of meshed electrode should be just slightly influenced by Coulomb force between mesh lines. It is very important to consider that current was expected to be equally distributed even in a large-area transparent heater when a voltage is applied. Therefore the car window would be integrally heated uniformly. To describe the Ohmic characteristic of these electrodes in a circuit intuitively, resistivity was converted into sheet resistance as follows^12^:

2 $${{\rm{R}}}_{{\rm{sh}}}(\Omega /\varUpsilon )={\rm{\rho }}(\Omega \cdot {\rm{cm}})/{{\rm{T}}}_{{\rm{film}}}$$

where R_sh_ is the sheet resistance, ρ is the measured sheet resistivity, and T_film_ is thickness of thin film. As seen from the red line of Fig. 3a, the sheet resistance of these electrodes decreased from 141 Ω/ϒ to 15.65 Ω/ϒ as the Ag thickness increased from 20 nm to 80 nm, and slightly increased to 17.9 Ω/ϒ when Ag is 100 nm. In general, it is observed that resistance characteristics of these meshed SAS electrodes are also highly depended on the thickness of middle Ag layers. From the result, it is expected that these square shaped electrodes will show a sheet resistance on the level of 10~10^2^ Ω/ϒ in a DC circuit where a value of 10 to 100 ohms means that the sheet resistance changes with the thickness of the silver.

Figure 3b is the optical transmittance of the meshed SAS electrode samples. When Ag-thickness is below 80 nm, the samples showed relatively high transmittance because the SAS multilayers themselves have some degree of transparency as shown in Fig. 2c. On the contrary, when the Ag thickness is greater than or equal to 80 nm, the as-deposited SAS multilayers become opaque, in this situation, the transmittance of mesh electrode can be calculated by^27^:

3 $${{\rm{T}}}_{{\rm{mesh}}}=100 \% \ast {(1-{\rm{FF}})}^{2}$$

Based on previous research, the electrical conductivity of mesh electrode is basically depending upon the filling factor (FF)^27^, which is defined by W/(W + S), where W is the width of the mesh and S stand for the mesh spacing. In this research, W is 15 μm and S is 400 μm. Here, the FF of our samples is calculated in the region of 3.61%, and the corresponding T_mesh_ is 92.9%. Table 1 shows the measured average optical transmittances (T_av_) of these samples. We can observe the T_av_ of mesh electrode with Ag thickness 80 nm and 100 nm are 92.02% and 91.74%, which is very close to the theoretically calculated data. And it was found that the transmittance is higher than other mesh electrodes^28^. For the comparison of SAS mesh and only Ag mesh (80 nm) structures, the transmittance and sheet resistance of only Ag mesh were measured to be about 90.33% and 16.3 Ω/ϒ. It is also important to note that the sheet resistance of only Ag mesh was degraded to 27.1 Ω/ϒ after 7 days since Ag was exposed to air and oxidized on the surface of Ag easily. T_av_ was calculated as follows^29^:

4 $${{\rm{T}}}_{{\rm{av}}}=\frac{\int {\rm{V}}({\rm{\lambda }}){\rm{T}}({\rm{\lambda }}){\rm{d}}{\rm{\lambda }}}{\int {\rm{V}}({\rm{\lambda }}){\rm{d}}{\rm{\lambda }}}\,(380{\rm{nm}} < {\rm{\lambda }} < 780{\rm{nm}})$$

where V(λ) is the luminous spectral efficiency, and T(λ) is the measured transmittance of thin films.

**Table 1:** Electrical and optical properties of SAS TMH and SAS multilayer.

	Mesh	Layer	Calculated Mesh
Sheet resistance (Ω/sq)	15.6	0.31	8.61
Transmittance (%)	91.74	1.35	92.92

Figure of merit (FOM) is an effective method to synthetically evaluate the electrical and optical properties of TCEs and FOM is defined as:

5 $${{\rm{\varphi }}}_{TC}=\frac{{T}_{av}^{10}}{{R}_{sh}}$$

where φ_TC_ is the figure of merit, T_av_ is the average optical transmittance, and R_sh_ is the sheet resistance^29^.

**Figure 4:**
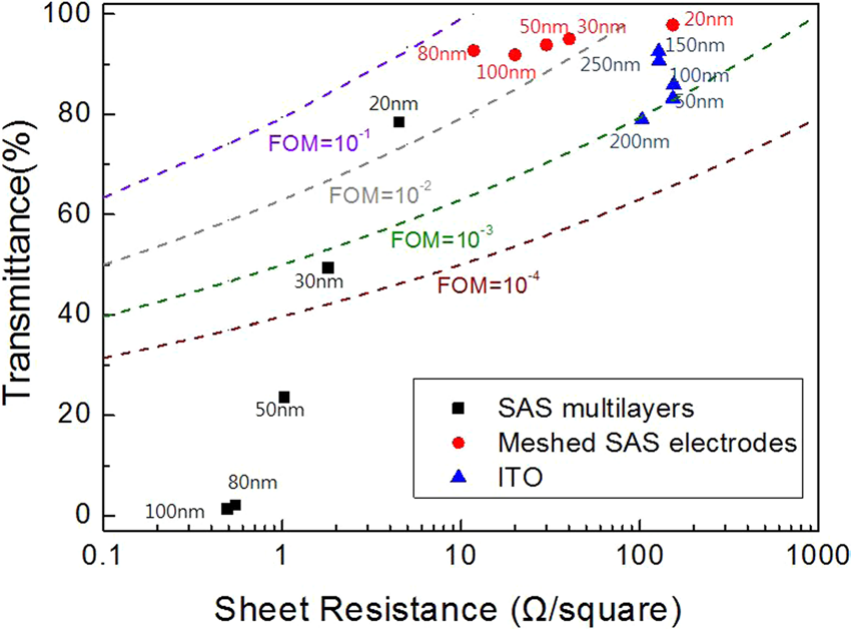
A review of different types of TCEs: as-deposited SAS multilayers, meshed SAS electrodes, and ITO layers. The X-axis shows sheet resistance values and the Y-axis shows average transmittance in visible region. Dashed lines in this figure shows the boundaries of FOM values which are 10^−4^, 10^−3^,10^−2^, 10^−1^ from bottom to top, respectively.

It is necessary to point out the merit of mesh processing, therefore, in here, the FOM values of as-deposited SAS, meshed SAS, and ITO thin films were summarized up in Fig. 4. As-deposited ITO-group with thickness varied from 50 nm to 250 nm showed T_av_ above 80%, but relatively high sheet resistance in the region from 103 Ω/ϒ to 155 Ω/ϒ without annealing treatment, therefore FOM values distributed in the region of 10^−3^ Ω^−1^~10^−2^ Ω^−1^. As-deposited SAS sample’s sheet resistance can be reduced to the level of 10^−1^ Ω/ϒ by increasing the thickness of Ag layer, however, the T_av_ dropped sharply, and nearly opaque when Ag layer is over 80 nm. Sheet resistance of meshed SAS also decreased sharply by increasing the thickness of Ag layer, but different form the as-deposited SAS samples, optical transmittance of meshed SAS layers is varied slightly, and the value of T_av_ is maintained to 91% when the thickness of Ag is above 80 nm because the transmittance of meshed electrode is basically depend upon the filling factor (FF) as explained before. It is observed that the FOM of SAS mesh electrodes reaches the high level region between 10^−1^ Ω^−1^ and 10^−2^ Ω^−1^, showing a great potential for transparent heaters. Additionally, when thickness of Ag layer is 20 nm, as-deposited SAS multilayer showed relatively high FOM value compared with meshed OMO electrode. This means that there seems no necessity to process the multilayers into mesh lines if thickness of Ag is thin enough, But meshing process should be an effective way to fabricate a high performance transparent electrode when the thickness of Ag is over 30 nm.

**Figure 5:**
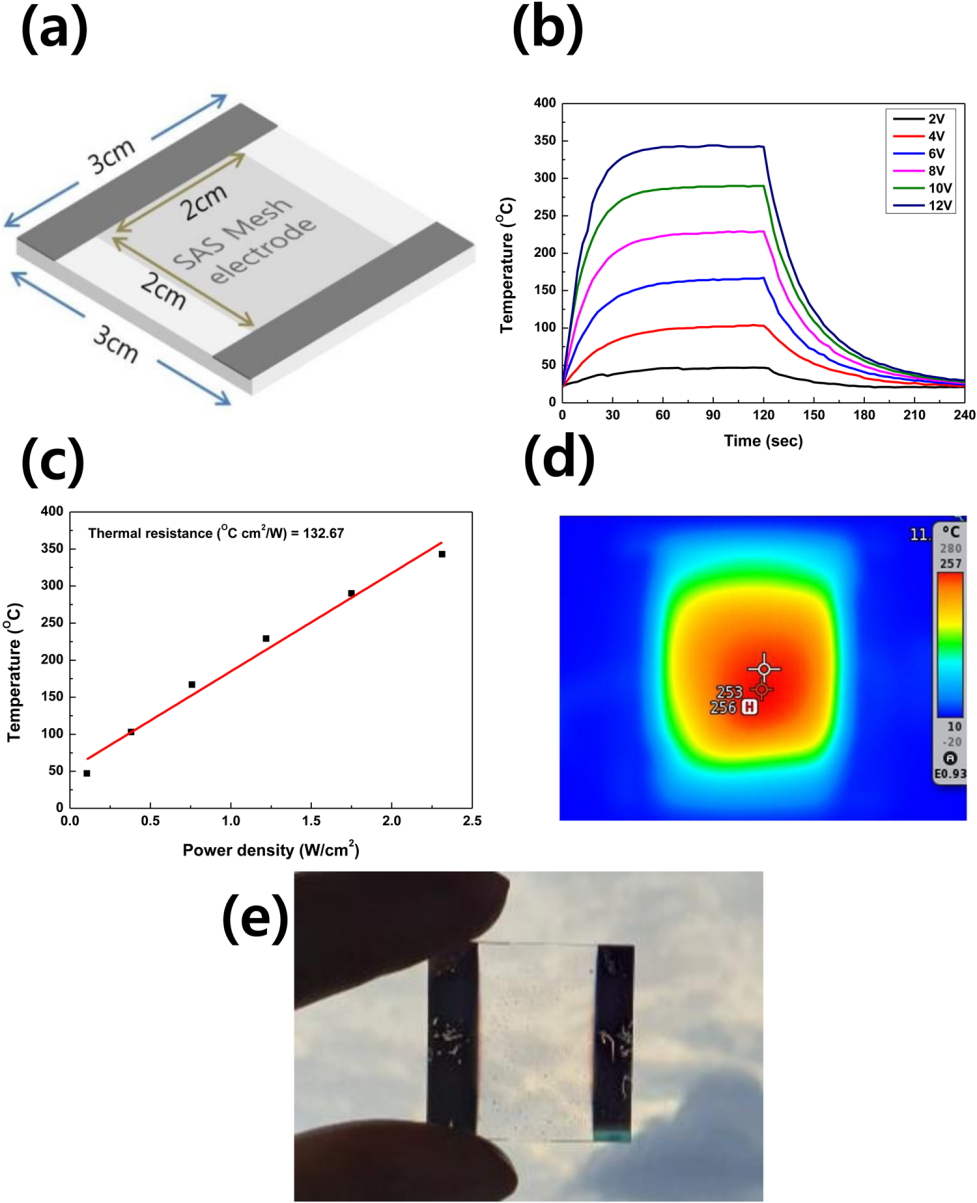
(**a**) Schematic of the SAS meshes fabricated for heating experiment, (**b**) Heating characteristic of SAS transparent mesh heater under different supply voltage of 2 V, 4 V, 6 V, 8 V, 10 V and 12 V, (**c**) Temperature versus power plot for the transparent heater, (**d**) Infrared camera imaging when the a 8 V voltage is applied for 30 second, (**e**) Specular transmittance of SAS meshes structure.

Afterwards, a 2 cm × 2 cm mesh electrode used for heating test was fabricated on a 3 cm × 3 cm glass substrate as shown in Fig. 5a, the dark grey part in this schematic is as-deposited S(80 nm)/A(100 nm)/S(80 nm) multilayer fabricated for connecting a power supply. Figure 5b showed the time-dependent temperature profiles of SAS TMH as changing applied DC voltage. After the voltage was applied, the temperature of SAS TMH was rapidly increased. As increasing applied DC voltage from 2 V to 12 V, the saturation temperature of SAS TMH was increased from 47 °C to 343 °C. The saturation temperature of the heater is linearly proportional to the applied power per unit area, as shown in Fig. 5c. In saturation temperature versus applied power plot, the slope is related with the thermal resistance. The thermal resistance is important parameter to determine heating property. The thermal resistance of SAS TMH shows 132.67 °C cm^2^/W. Figure 5d showed IR camera image for the heat generation of SAS TMH. The heating property was calculated by Joule’s law. In case of applying constant DC voltage, the Joule’s law can be expressed as following:

$${\rm{Q}}=VIt=\frac{{V}^{2}}{R}\times t$$

where Q is the heat produced, V is the applied DC voltage, R is the resistance, and t is the working time. Therefore, the saturation temperature of the heater was proportional to the applied DC voltage and the reverse sheet resistance.

**Figure 6:**
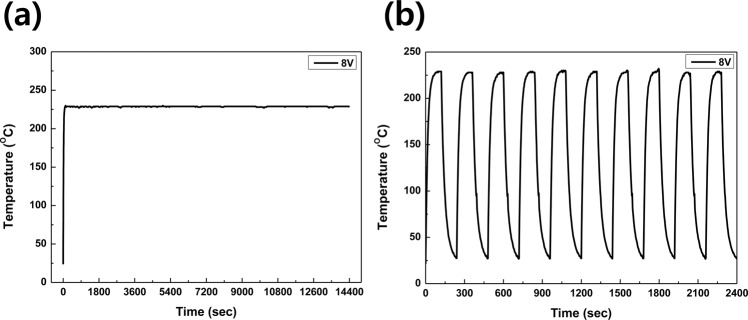
Heater performance of the SAS transparent mesh heater. (**a**) Heating experiments showing peak temperature as a function of time for investigation of long-term working stabilities of SAS transparent mesh heater, (**b**) The heating cycle test by switching the voltage (0 V and 8 V) every 120 s.

As the main pathway of electrical conduction, Ag layer is suggested to be well protected against oxidation^20^. In meshed SAS multilayer structure, Ag middle layer was encapsulated by both the bottom and top SIZO layer, in here, oxidation resistance tests of the meshed SAS electrode were carried out in two ways: To investigate the long-term working stabilities of the SAS TMH, constant voltage of 8 V was supplied for 4 hrs. The SAS TMH exhibited stable heating property up to 229 °C, as shown in Fig. 6a. The performance repeatability of the SAS transparent mesh heater was investigated systematically by the heating cycle test for 10 times. Figure 6b shows the time-dependent temperature of SAS transparent mesh heater when the applied voltage was repeatedly switched between 0 V and 8 V every 120 s. The temperature sharply increased from room temperature to 228 °C after a voltage was applied repeatedly without degrading heating performance.

**Figure 7:**
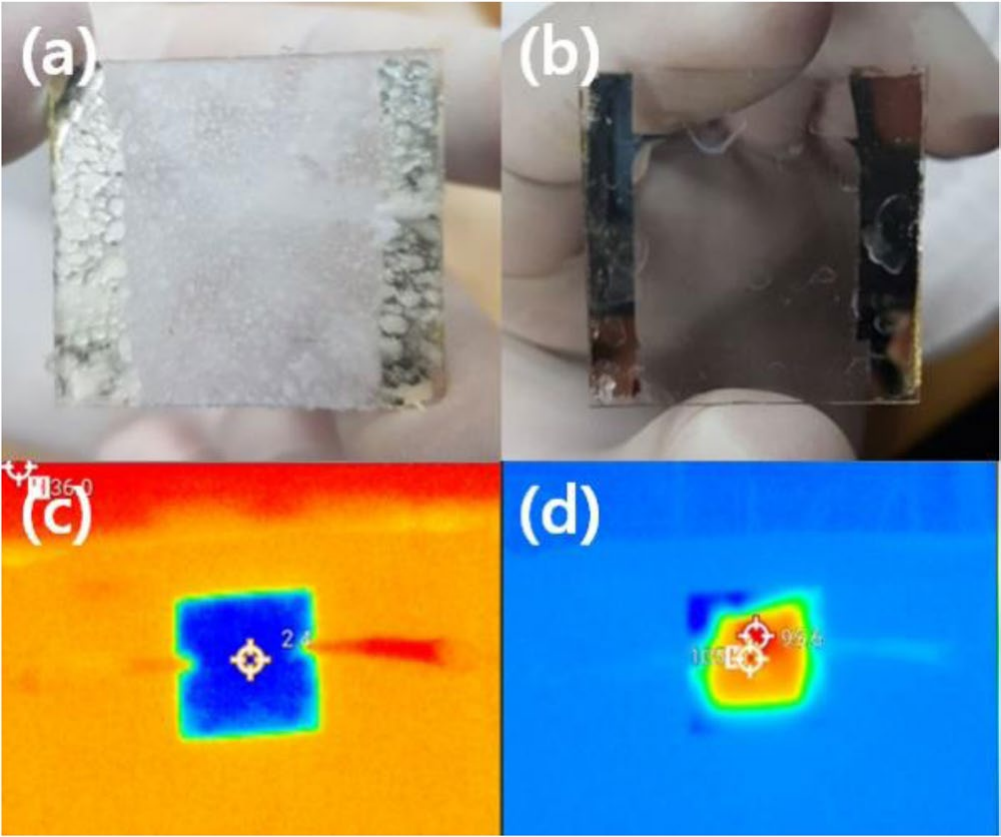
Performance of SAS transparent mesh as defrosting glass substrate. (**a**) before and (**b**) after operation of the film heater with a driving voltage of 10 V. The infrared camera images of defrosting test as shown in (**c**) before and (**d**) after.

Figure 7 demonstrates the defrosting test result of the SAS TMH before and after applying voltage. To generate frost completely on SAS TMH surface, the sample was refrigerated by using dry ice. Defrosting test was performed by applying DC voltage of 10 V. After applying voltage, the frost on SAS TMH was rapidly removed (1.5 sec) as shown in Fig. 7(a),(b). The infrared camera image of this result was shown in Fig. 7(c),(d). This indicates that it is possible for SAS TMH to be used as frost remover device in smart windows for buildings and automobiles.

## Discussion

As a summary, by fabricating as deposited SAS multilayers with different thickness of middle Ag layers, it is clearly observed that thickening the middle Ag layer could effectively improve both mobility and carrier concentration in as-deposited SAS multilayer and improve electrical conductivity, but metal-like optical properties were observed if the thickness of middle Ag is over 30 nm. Taking the use of mesh processing, as-deposited SAS multilayers were fabricated into micron-scale mesh lines. Meshed electrodes showed high average transmittance over 90%, and low sheet resistance in the region from 141Ω/ϒ to 15.65 Ω/ϒ, which shows the necessity of mesh processing. We summarized FOM values of different types of TCEs, such as ITO, SAS multilayers, and meshed SAS, compared with commercial ITO and as-deposited SAS electrode. Meshed SAS electrode shows relatively high FOM values, benefit from their dramatically improved optical transmittance. Afterwards, heating experiment is carried out by using a meshed S(80 nm)/A(100 nm)/S(80 nm) transparent heater, showing the benefit from its high electric conductivity. When applied voltages is 10 V, temperature quickly raised from room temperature to 130^o^C in just 5 seconds, then reached to maximum of 250^o^C in tens of seconds, which showed very efficient heating characteristic. Also excellent Ag oxidation resistance of meshed SAS electrode were also observed. On both hand of performance and stability, meshed SAS electrode shows potential for the fabrication of high conductive TCEs used in transparent heaters for electric vehicle application.

## Methods

### Deposition of SIZO/Ag/SIZO multilayers

Amorphous SIZO (a-ISO) films which were used as bottom/top oxide layers were prepared on a glass (Corning 1737) substrate by RF magnetron sputtering at room temperature. The sputtering conditions of a-SIZO layers, which are base pressure, working pressure, Ar gas flow rate, sputtering rate, and sputtering power, were 5×10^−6^ torr, 4.0 mTorr, 30 sccm, 3.467 nm/min, and 30 W. Amorphous SIZO films were deposited by using a ceramic Si–In-Zn–O target (Lab-made). The middle Ag film which was sandwiched between two outer oxide layers was also grown by DC method at normal temperature, under pure Ar plasma gas pressure of 4.0 ×10-3 Torr, sputtering power 20 w, the sputtering rate is 4.1 Å/s under this condition, the Ag layer thickness was changed systematically, ranging from 20 nm to 100 nm.

### Meshing pattern processing

Figure 2(a) illustrates the mesh-patterning process of sputtered SAS multilayer films by using photo resist processing, the mask pattern was designed as: the square mesh grid line width is 15 μm, and the spacing between the grids was 400 μm, to enhance the connection between these grid lines. The same pattern was printed once again angularly. The mesh line SAS electrode fabricated with this crossed pattern is shown in Fig. 2(b).

### Measurement of electrical and optical properties

The visible and IR transmittance were analyzed by UV–VIS optical spectrometer (Cary 5000 UV-Vis-NIR, Agilent), the electrical properties was analyzed by Hall-effect measurement (HMS-3000). The SAS thickness was confirmed exactly by using scanning electron microscope (SEM, JSM-7610F, JEOL Company, Supplementary Fig. 1).

## Supplementary information

Supplementary Figure 1.
