# Supplementary material for: Transparent heater with meshed amorphous oxide/metal/amorphous oxide for electric vehicle applications
Source: Sci Rep. 2020 Jun 16;10:9697. doi: 10.1038/s41598-020-66514-8 (PMC7297967; doi:10.1038/s41598-020-66514-8)
Supplement: Supplementary file 1 — Supplementary Figure 1. [file 41598_2020_66514_MOESM1_ESM.docx]

Supplementary Information

Transparent heater with meshed amorphous oxide/metal/amorphous oxide for electric vehicle applications

Sang Yeol Lee and Jin Young Hwang

Supplementary Figures


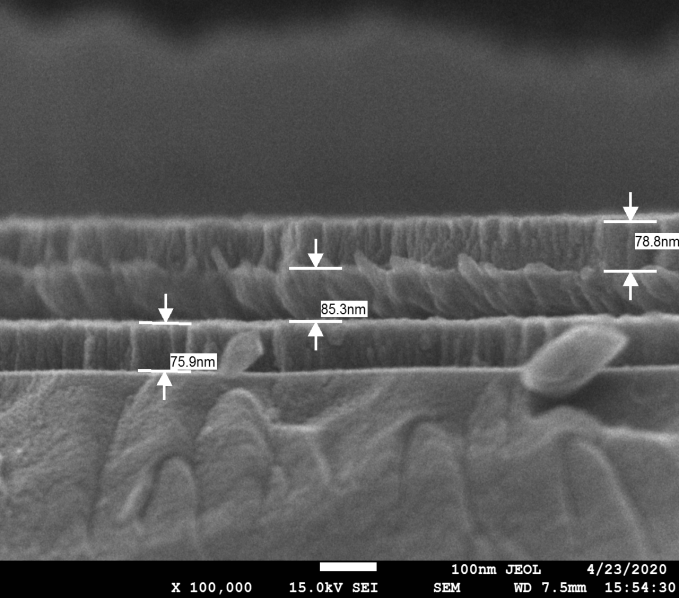

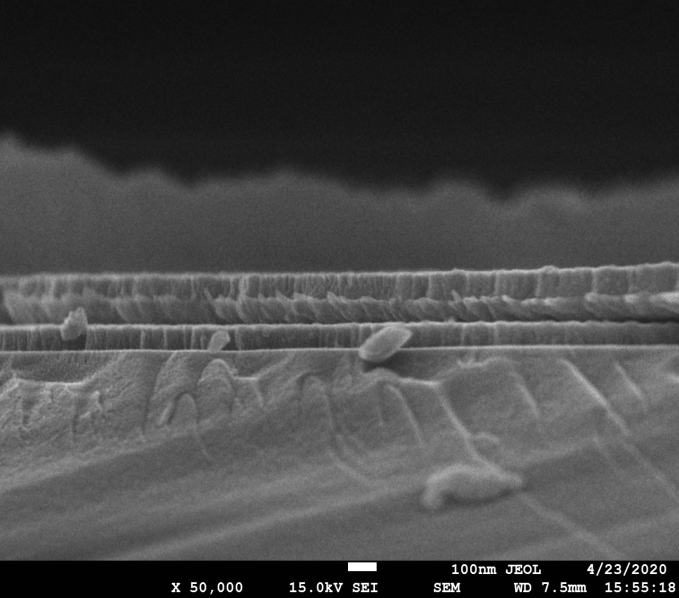


(a)


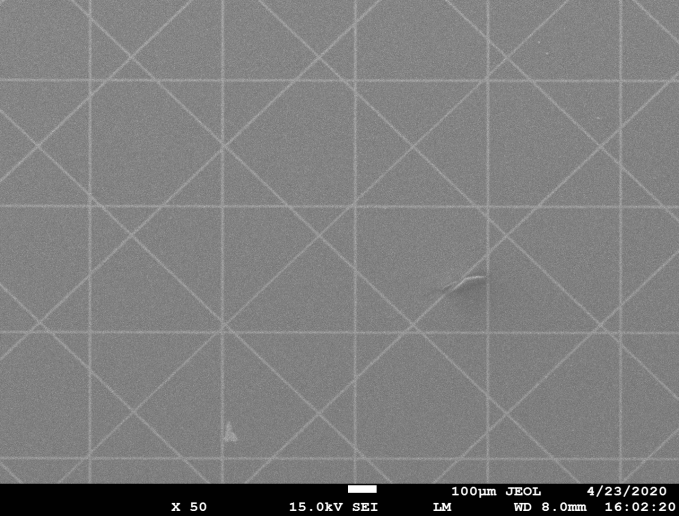

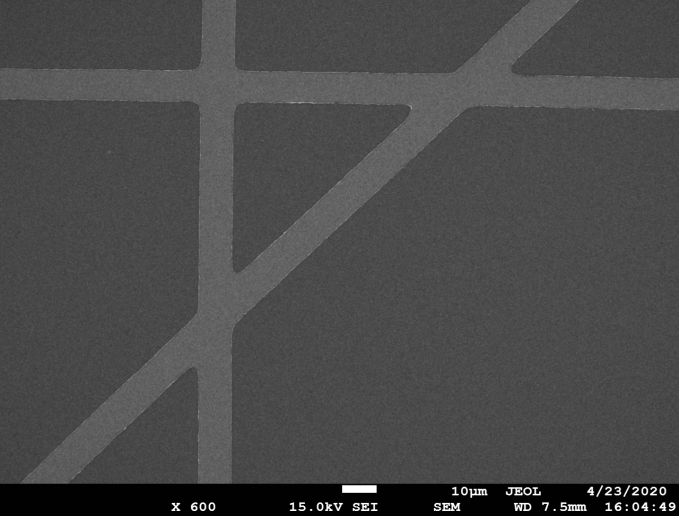


(b)

Supplementary Figure 1. SEM measurements show in (a) the thickness and (b) the surface image.
